# CRISPR whole-genome screening identifies new necroptosis regulators and *RIPK1* alternative splicing

**DOI:** 10.1038/s41419-018-0301-y

**Published:** 2018-02-15

**Authors:** Marinella G. Callow, Colin Watanabe, Katherine E. Wickliffe, Russell Bainer, Sarah Kummerfield, Julie Weng, Trinna Cuellar, Vasantharajan Janakiraman, Honglin Chen, Ben Chih, Yuxin Liang, Benjamin Haley, Kim Newton, Michael R. Costa

**Affiliations:** 1 Department of Discovery Oncology, Genentech, Inc., 1 DNA Way, South San Francisco, CA 94080 USA; 2 Department of Bioinformatics and Computational Biology, Genentech, Inc., 1 DNA Way, South San Francisco, CA 94080 USA; 3 Department of Physiological Chemistry, Genentech, Inc., 1 DNA Way, South San Francisco, CA 94080 USA; 4 Department of Molecular Biology, Genentech, Inc., 1 DNA Way, South San Francisco, CA 94080 USA; 5 Department of Biochemical and Cellular Pharmacology, Genentech, Inc., 1 DNA Way, South San Francisco, CA 94080 USA; 6 Present Address: Department of Molecular Biology, Princeton University, Lewis Thomas Laboratory, Washington Road, Princeton, NJ 08544 USA

## Abstract

The necroptotic cell death pathway is a key component of human pathogen defense that can become aberrantly derepressed during tissue homeostasis to contribute to multiple types of tissue damage and disease. While formation of the necrosome kinase signaling complex containing RIPK1, RIPK3, and MLKL has been extensively characterized, additional mechanisms of its regulation and effector functions likely remain to be discovered. We screened 19,883 mouse protein-coding genes by CRISPR/Cas9-mediated gene knockout for resistance to cytokine-induced necroptosis and identified 112 regulators and mediators of necroptosis, including 59 new candidate pathway components with minimal or no effect on cell growth in the absence of necroptosis induction. Among these, we further characterized the function of PTBP1, an RNA binding protein whose activity is required to maintain RIPK1 protein abundance by regulating alternative splice-site selection.

## Introduction

Multiple pathways in multicellular organisms have evolved to induce and execute cell death in defective, infected, or extraneous cells, and dysregulation of numerous steps in each pathway contribute to diseases and tissue damage^1,2^. Necroptosis is a caspase-independent form of programmed necrosis that mediates defense against viral pathogen infection, particularly when caspase activation is blocked, by directly lysing infected cells and releasing damage-associated molecular patterns (DAMPs) to initiate an inflammatory response^3,4^. While clinical biomarkers of necroptosis have not yet been widely employed in human studies, necroptosis is pathologically activated in certain mouse models of a variety of diseases and disorders involving tissue injury, degeneration, and inflammation^5,6,7^. Notably, genetic or pharmacologic inhibition of the necroptosis mediators RIPK1 kinase activity, RIPK3, or MLKL demonstrates therapeutic benefit in mouse models of inflammatory bowel disease, ischemia–reperfusion injury, drug-induced liver injury, acute kidney injury, retinal detachment, chronic dermatitis, atherosclerosis, and amyotrophic lateral sclerosis (ALS)^6,7^. However, RIPK1 enzymatic activity and *Ripk3* can also mediate apoptosis in certain contexts, and *Mlkl* deficiency is less protective than *Ripk3* deficiency or catalytically inactive RIPK1 in some tissue injury and inflammation models^8^.

Necroptosis is induced when caspase-8 is inhibited and either a TNF family death-domain receptor, Toll-like receptor TLR3 or TLR4, or the virus sensing adaptor protein ZBP1 is triggered. RIPK1 (recruited to death receptors), TRIF (recruited to TLR3 and TLR4), and ZBP1 are RHIM domain-containing proteins that activate the kinase RIPK3 through homotypic interaction with its RHIM domain^9,10^. Autophosphorylated RIPK3 in turn phosphorylates the pseudokinase MLKL to induce a conformational change, homo-oligomerization, and translocation to the plasma membrane to promote membrane permeabilization and cell lysis^11–13^. However, necroptosis is actively suppressed during development and in many adult tissues, such that in most cell types TNFR1 activation does not result in cell death. In a context-dependent manner, RIPK1 function is regulated at multiple checkpoints by association of RIPK1 with different binding partners in alternative complexes^9,14,15^. In the most completely characterized example of pathway activation, engagement of TNFR1 by the cytokine TNFα assembles complex-I at the cell membrane by recruiting RIPK1, TRADD, TRAF2/5, and the E3 ubiquitin ligases cIAP1/2 and LUBAC that ubiquitylate RIPK1 and other components of complex-I^9,14-16^. In a kinase-independent manner, ubiquitylated RIPK1 recruits and activates IKK and TAK1-TAB2/3 complexes, stimulating NF-κB, JNK, p38, and ERK pathways to transcriptionally activate anti-apoptotic and pro-inflammatory genes, including CFLAR (cFLIP) encoding the catalytically inactive homolog of caspase-8. Under conditions where RIPK1 is not efficiently ubiquitylated, complex-I is unstable, and RIPK1 dissociates to form cytoplasmic complex-II with FADD and caspase-8 in a RIPK1 kinase-dependent manner. RIPK1 ubiquitylation can be restricted by cIAP inhibition or by the deubiquitylase activity of CYLD, which is recruited to the complex-I through the LUBAC-binding protein SPATA2^17-22^. Activated caspase-8 within complex-II triggers apoptosis by cleaving executioner caspases. However, if caspase-8 expression is limited or its activity is blocked by pan-caspase inhibitors such as zVAD-fmk, then complex-II can further develop to form the necrosome complex as RIPK1 recruits and activates RIPK3, leading to MLKL-dependent necroptosis^3,9,10,15^.

Despite current knowledge of the RIPK1 and necroptosis signaling pathways, many aspects of their regulatory mechanisms remain to be elucidated, including ubiquitin editing dynamics, protein complex intracellular trafficking, and membrane channel interactions. Expression levels of RIPK1, RIPK3, and MLKL correlate with sensitivity to necrosis in some mouse models and human disease tissues^2^, and defining their transcriptional and post-transcriptional control could help verify a primary causal relationship. To discover new pathway components that might ultimately reveal new therapeutic intervention strategies or predictive biomarkers for inhibitors in preclinical development, we performed a whole-genome CRISPR screen selecting for necroptosis resistance. This screen successfully identified 59 new candidate regulatory genes previously implicated in diverse cellular functions that, similar to RIPK1, RIPK3, and MLKL, display minimal or no requirement for normal cell proliferation. We further defined the screen hit *Ptbp1* as a determinant of RIPK1 protein expression levels by demonstrating that it represses alternative splicing to a cryptic exon that induces a frame shift in the *Ripk1* transcript.

## Results

### Genome-wide CRISPR screening identifies new necroptosis pathway regulators

We developed a screen in L929 mouse fibroblast cells to discover new genes whose products mediate or modulate the induction and execution of necroptosis stimulated by recombinant TNFα protein in the presence of the pan-caspase inhibitor zVAD-fmk^23^. To do so, we generated a transgenic cell line stably expressing Cas9 and validated that co-expression of a sgRNA targeting *Mlkl* reduced MLKL protein to undetectable levels and conferred nearly complete resistance to necroptosis within 8 days (Figure S1). We used this cell line to screen a novel whole-genome sgRNA library evenly partitioned in nine pools targeting an average of 2203 genes, with 93% of genes targeted by eight sgRNAs (Figure S2). sgRNA sequences were selected using an algorithm that designs sgRNAs and prioritizes them at the gene level on the basis of the number of splice isoforms targeted, proximity to the 5′ end of the coding region, and the number of predicted off-target sites (see Materials and methods). Three of the library pools were screened individually at 1000-fold sgRNA representation with a target multiplicity of infection (MOI) of 0.3–0.6. The remaining six libraries were screened as three combinations of two pools at 500-fold sgRNA representation and MOI 0.6, after determining that these parameters did not significantly reduce the screen hit detection power (Table S7). Each library contained at least one sgRNA targeting *Mlkl* as a positive control, as well as containing a common set of 47 non-targeting control (NTC) sgRNAs. For each of the six screens, three replicate samples were transduced and independently maintained at the indicated sgRNA representation through three passages over 10 days to allow for gene knockout. A cell number that maintained 500–1000-fold sgRNA representation was collected for a reference time point 2 days after transduction, and puromycin was added to an equivalent number of cells to select for sgRNA integrations starting 3 days after transduction. Ten days after transduction, ~1.5 × 10^8^ cells per replicate were treated with TNFα and zVAD-fmk to achieve 99.0–99.9% cell killing by necroptosis, and resistant cells were harvested 5 days later. In parallel, untreated cultures were maintained at the indicated sgRNA representation through an additional passage and collected at the same time as the treated samples.

**Fig. 1:**
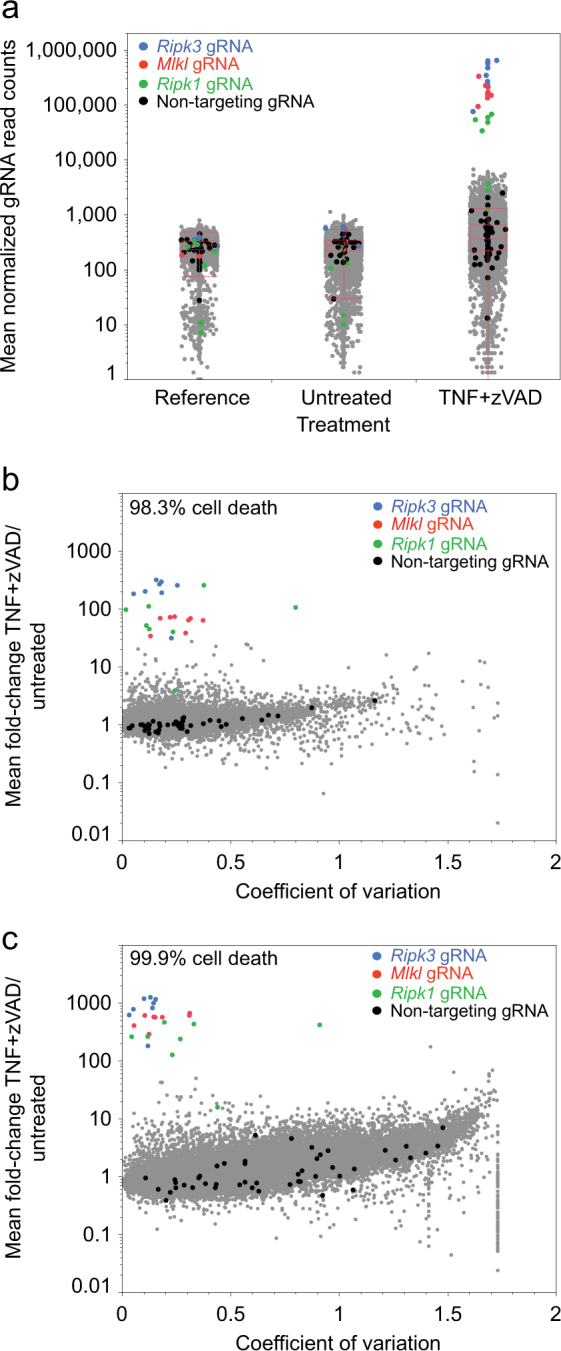
Positive selection for necroptosis resistance of L929 cells enriches for sgRNAs targeting necrosome component genes. Mean values of all three replicates for each sgRNA in the Library 1 screen are shown, with non-targeting, *Ripk1*, *Ripk3*, and *Mlkl* sgRNAs marked with the indicated colors. **a** Normalized sgRNA read count distributions for day 2 reference, untreated (day 15), and TNFα+zVAD-treated samples (day 15). **b**,** c** Comparison of sgRNA read count mean fold-change and variation between replicate samples for two screens after necroptosis induction producing 98.3% (**b**) or 99.9% (**c**) cell death

Genomic DNA (gDNA) extracted from the reference, treated, and untreated cell collections was used in an amount that maintained the sgRNA representation for PCR amplification of the sgRNA sequences. Read counts for the individual sgRNAs were determined by next-generation sequencing (NGS) of the PCR amplicons, after correcting for library sequencing depth and scaling the median sgRNA read count number across libraries (Table S1; see Materials and methods). The very strong level of positive selection achieved in the screens with TNFα and zVAD-fmk provides a large assay window of over 1000-fold sgRNA enrichment, although it also produces a relatively high level of sgRNA read count variation between some replicates of only the treated samples, presumably due to stochastic differences in survival of small numbers of cells with sgRNAs that do not confer resistance (Fig. 1 and S3). The gCrisprTools software^24^ was used to summarize sgRNA-level signals into gene-level estimates (Rho scores) of the significance of sgRNA enrichment in the treated samples compared to the time-matched untreated controls, or of sgRNA depletion in the untreated controls compared to the early reference time point samples. This analysis effectively prioritizes genes targeted by sgRNAs that are consistently enriched across replicate treated samples relative to their paired untreated controls. A total of 112 genes in the genome-wide screens show Rho scores less than 10^−9^ and are considered further here (Table S2 and Figure S4a).

**Fig. 2:**
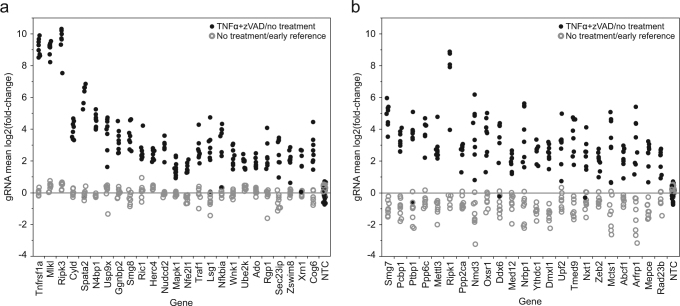
Necroptosis resistance screen hit identification and classification. Read count fold-change for all sgRNAs targeting screen hits with gene-level Rho scores for enrichment after necroptosis induction <10^−9^, and Rho scores for depletion without TNFα+zVAD treatment greater than 0.05 (**a**) or between 0.05 and 10^−6^ (**b**). Solid dots, fold-change with TNFα+zVAD treatment compared to untreated; open circles, fold-change without treatment compared to day 2 reference; NTC non-targeting control sgRNAs

Among the samples treated with TNFα and zVAD-fmk, we observe the highest level of sgRNA enrichment (up to 1260-fold) among sgRNAs targeting the gene that encodes TNF receptor *Tnfrsf1a* (*Tnfr1*) and the three canonical components of the necrosome, *Ripk1*, *Ripk3*, and *Mlkl* (Fig. 2). Almost all sgRNAs targeting the genes encoding the deubiquitylase CYLD and its recently identified binding partner, SPATA2, are also strongly enrichment, consistent with the reported roles of CYLD and SPATA2 in deubiquitylating RIPK1 and enhancing its kinase activity in complex-I and the necrosome^17-21^. sgRNAs targeting *Tnfrsf1a*, *Mlkl*, *Ripk3*, *Cyld*, and *Spata2* were not significantly depleted in the time-matched untreated samples in their respective screens, whereas most sgRNAs targeting *Ripk1* were weakly depleted, suggesting that RIPK1 may also function independent of TNFα signaling to promote cell proliferation or survival. This RIPK1 pro-survival function may be related to the perinatal lethality of *Ripk1*-deficient mice caused by induction of apoptosis and necrosis^25-27^. Many of the 112 screen hit genes show even greater levels of sgRNA depletion in the absence of necroptosis induction, including known essential genes such as components of the ribosome (*Rpl8*, *Rpl13a*) and chaperonin containing TCP1 complex (*Cct2*), possibly indicating that some mechanisms of generally slowing cell growth can delay necroptosis. In an attempt to distinguish screen hits more specifically regulating necroptosis, we classified genes based on arbitrary minimum Rho scores for depletion in untreated cells (Figure S4b). Twenty-five genes, including *Tnfrsf1a*, *Mlkl*, *Ripk3*, *Cyld*, and *Spata2*, have a depletion Rho score greater than 0.05, suggesting no effect on cell viability or proliferation (Fig. 2a). This group includes *N4bp1*, which has a level of sgRNA enrichment after necroptosis induction similar to *Cyld* and *Spata2*, but has not previously been reported to function in necroptosis. An additional 23 hits have Rho scores between 0.05 and 10^−6^ for sgRNA depletion in untreated cells, and this class includes *Ripk1* and *Ptbp1* (Fig. 2b). A third tier of 17 hits possess Rho scores between 10^−6^ and 10^−8^ (Table S3).

### PTBP1 regulates *Ripk1* splicing and expression

**Fig. 3:**
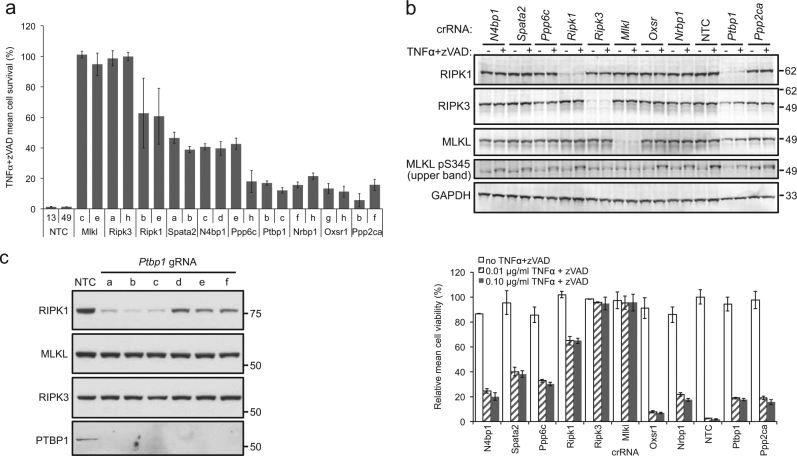
Screen hit validation with transfected synthetic crRNAs in L929 cells. **a** Necroptosis resistance levels for cells transfected with annealed crRNA:tracrRNA and treated with TNFα+zVAD, compared to untreated cells. Error bars represent standard deviation (*N* = 4). **b** Protein levels (upper panel) and cell survival (lower panel) after transfection with crRNA:tracrRNA duplexes, with or without TNFα+zVAD treatment as indicated. GAPDH serves as a control for protein loading, and indicates lower loading of *Ptbp1* crRNA-treated samples. Error bars represent standard deviation (*N* = 4). **c** Protein expression levels as measured by western blotting after infection with *Ptbp1* or non-targeting control sgRNAs. Error bars represent standard deviation (*N* = 4). Molecular weight marker sizes are indicated in Kd to the right of western blots (**b, c**)

To confirm screen hits, we developed a 96-well format assay for arrayed synthetic crRNAs. crRNAs containing the same targeting sequence as those in the pooled sgRNA libraries were synthesized, annealed with synthetic tracrRNA, and transfected into the L929 Cas9 stable cell line. After 7 days of cell growth to allow for gene knockout, we induced necroptosis with TNFα and zVAD-fmk before measuring cell viability. crRNAs targeting *Mlkl* and *Ripk3* conferred nearly complete resistance to necroptosis, whereas ~60–65% of cells transfected with crRNAs targeting *Ripk1* were rendered insensitive to the treatment (Fig. 3a, b). Consistent with the screen results, crRNAs against all seven of the novel hit genes tested conferred significant but lower levels of necroptosis resistance, ~25–40% for *Spata2* and *N4bp1* (Fig. 3a, b). None of the crRNAs targeting these novel hits reduced cell viability in the absence of necroptosis induction by more than ~15%, although we note that in principle some of these genes might influence cell viability in a manner that is undetectable in a short-term assay (Fig. 3b).

We investigated whether any of these seven new regulators of necroptosis acted by altering expression of necrosome components. crRNA-mediated knockout of *Ptbp1* specifically reduced protein levels of RIPK1, but not RIPK3 or MLKL, and this activity is not dependent on stimulation of necroptosis (Fig. 3b, c). crRNAs targeting the other six candidate genes identified in the screen did not clearly affect levels of RIPK1, RIPK3, or MLKL proteins, and did not impair phosphorylation of MLKL at Ser345 upon TNFα and zVAD-fmk stimulation (Fig. 3b). However, we note that the residual RIPK1 protein after *Ripk1* or *Ptbp1* crRNA transfection can also mediate detectable MLKL phosphorylation, consistent with their conferring only partial necroptosis resistance, so we cannot definitively conclude that the screen hits act downstream of MLKL activation.

**Fig. 4:**
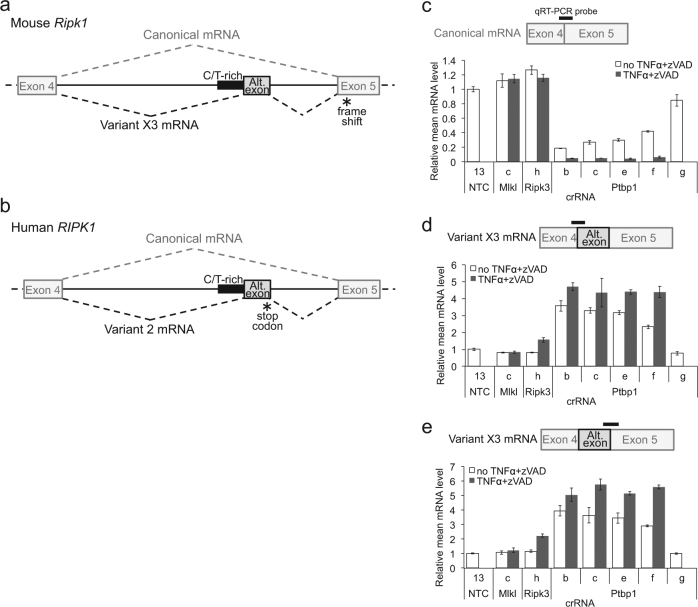
*Ptbp1* regulation of *Ripk1* splicing and mRNA expression. **a**,** b** Schematic diagram of mouse (**a**) and human (**b**) *RIPK1* gene splicing between canonical exons 4 and 5, and the alternative exon (Alt. exon). **c**–**e** *Ripk1* mRNA expression levels, detected by qRT-PCR using primer and probe sets spanning exons as indicated by the bars, after L929 cell transfection of the indicated crRNAs and treated with or without TNFα+zVAD. Expression levels are normalized to *Actb* mRNA detected in the same sample. Error bars represent standard deviation (*N* = 2). Canonical mRNA, mouse RefSeq Accession NM_009068.3 and human NM_003804.4; Variant X3 mRNA, XM_011244295.2; Variant 2 mRNA, NM_001317061.1; Alt.exon, alternative exon; C/T-rich, putative PTBP1-binding polypyrimidine tracts; *, stop codon introduced by alternative splicing

Since PTBP1 is known to control alternative mRNA splicing of certain genes^28–30^, we further explored a potential role for splicing in the mechanism of action of PTBP1 in maintaining RIPK1 protein expression. Multiple *Ptbp1* crRNAs that reduce PTBP1 protein levels and confer necroptosis resistance also specifically decrease RIPK1 protein abundance (Fig. 3c). Steady-state levels of *Ripk1* mRNA are reduced by these active *Ptbp1* crRNAs, but not inactive Ptbp1_g crRNA, independent of necrosome activation (Figure S5). The National Center for Biotechnology Information Reference Sequence Database has annotated the mouse *Ripk1* transcript variant X3 (RefSeq Accession XM_011244295.2) with an alternative exon between canonical exons 4 and 5 that is spliced to exon 5 to generate a frame shift and premature stop codon (Fig. 4a and S6). Immediately upstream of the 3′ splice site at the alternative exon lies a 139-nt region containing 80% C/U content, characteristic of the polypyrimidine tracts bound by PTBP1 protein to repress cryptic exons^29^ (Figure S6). Using qRT-PCR primers that span the splice junctions of exon 4, exon 5, and the alternative exon, we found that *Ptbp1* knockout enhances splicing of exons 4 and 5 to the alternative exon and reduces the more prevalent splicing of exon 4 directly to exon 5 (Fig. 4c–e, S7c, and S7d). qRT-PCR with primers internal to exon 5 (Figure S5b) or exon 11(data not shown) demonstrate that total levels of *Ripk1* mRNAs are potently diminished by *Ptbp1* crRNAs, suggesting that the enhanced alternative splicing leads to nonsense-mediated decay (NMD) of transcripts that contain the premature termination codon. We conclude that PTBP1 acts as a repressor of a *Ripk1* cryptic exon that is deleterious to RIPK1 protein expression.

**Fig. 5:**
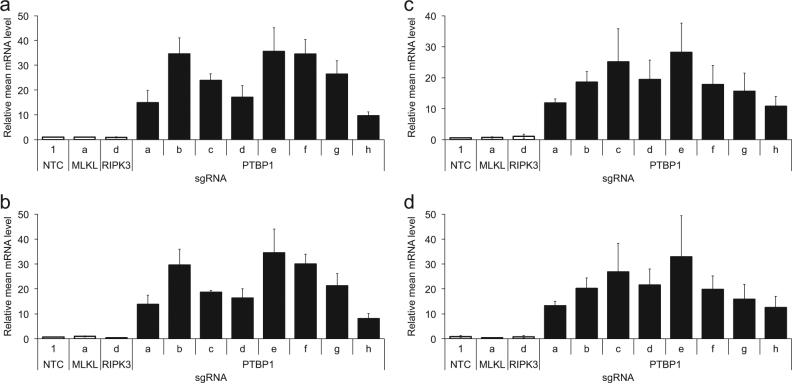
Human *PTBP1* regulation of *RIPK1* splicing. *RIPK1* mRNA expression levels, detected by qRT-PCR using primer and probe sets spanning exon 4 and the alternative exon (**a**, **c**) or the alternative exon and exon 5 (**b**,** d**), after HT-29 (**a**, **b**) or COLO 205 (**c**,** d**) cell infection with the indicated sgRNAs. Expression levels are normalized to *RPLP0* mRNA detected in the same sample. Error bars represent standard deviation (*N* = 3)

The cryptic exon and polypyrimidine tract are conserved in human *RIPK1*, and transcript variant 2 (RefSeq Accession NM_001317061.1) similarly harbors this alternative cassette exon that contains a premature termination codon (Fig. 4a and S6). In two human colorectal adenocarcinoma lines, HT-29 and COLO 205, that are sensitive to necroptosis induced by treatment with TNFα, zVAD-fmk, and cIAP inhibitor BV6, multiple sgRNAs targeting *PTBP1* enhance inclusion of the *RIPK1* alternative exon (Fig. 5a–d). However, the proportion of the alternative splice isoform relative to the canonical splice product that excludes the alternative exon remains <10% in these cell lines (Figures S7a and S7b), whereas more than half of the *Ripk1* transcripts can contain the alternative exon in mouse L929 cells with *Ptbp1* knockout (Figures S7c and S7d). Consequently, we observe minimal or no reduction in *RIPK1* canonical splicing of exon 4 to exon 5 (Figure S7e and S7f), or in resistance to necroptosis (Figure S7g and S7h), upon *PTBP1* knockout in the human colorectal cancer cell lines.

While necroptosis is not known to function in normal development or tissue homeostasis, necrosome components are expressed in a broad range of tissue types and developmental stages. Our survey of tissue RNA by qRT-PCR reveals detectable, though relatively low, levels of the *RIPK1* alternative splice product in many human (Figure S8a) and mouse (Figure S8b and S8c) tissues, with higher levels in brain. During embryonic neural development, PTBP1 protein levels decline in neural progenitor cells to initiate an alternative splicing program that mediates neuronal differentiation and *PTBP2* expression^28–30^. Comparing mouse cerebral cortex and cerebellar tissues and their precursors, we find that the level of *Ripk1* alternative splice isoform is higher in postnatal compared to embryonic stages, when *Ptbp1* expression is lower (Figure S8c). To further substantiate this reciprocal relationship, we examined neuronal differentiation of human induced pluripotent stem cells (iPSC). During conversion of the human neural stem cells (NSC) to differentiated neurons, *PTBP1* mRNA levels decline to a minimum at day 9, whereas expression *PTBP2* and the *RIPK1* alternative splice product increase to a maximum at day 12 (Figure S9a and S9b). Although *RIPK1* alternative splicing seems to reflect *PTBP1* expression levels, the canonical *RIPK1* transcript lacking the alternative exon remains the predominant splice isoform and does not clearly diminish during neuronal differentiation (Figure S9a).

## Discussion

We have executed and rigorously analyzed a positive selection screen of all targetable mouse protein coding sequences for necroptosis resistance using CRISPR/Cas9 gene knockout technology, employing an effective custom sgRNA library. The screen identified TNF receptor *Tnfrsf1a* and known necrosome components *Ripk1*, *Ripk3*, and *Mlkl* by strong sgRNA enrichment. More moderate sgRNA enrichment identified RIPK1 deubiquitylase CYLD as a positive regular of necroptosis, consistent with its facilitating RIPK1 translocation from the TNFR1 complex to the necrosome^17,22^, as well as SPATA2 which has subsequently been reported to recruit CYLD to the TNF receptor complex^18–21^. Most importantly, we discovered 20 new genes as necroptosis mediators that show no significant requirement for cell viability in the absence of necroptosis induction, as well as 39 novel necroptosis modulators that are only weakly required for normal cell viability (Table S3). It will be important to determine whether the subpopulation of cells resistant to necroptosis after knockout of these genes can be molecularly defined. Transfection of crRNA:tracrRNA synthetic oligonucleotides confirmed seven novel screen hits that were tested for necroptosis resistance of L929 cells (no other hits were tested).

Previously Hitomi et al^31^. performed a similar screen for survival of L929 cells treated with zVAD-fmk to induce necroptosis, however this screen utilized siRNAs for gene knockdown and was dependent on autocrine TNFα signaling. Their 432 confirmed screen hits include nine genes that were also identified in our screen: expected genes *Tnfrsf1a*, *Ripk1*, *Cyld*, and *Spata2*, and unanticipated genes *Ptbp1*, *Pcbp1*, *Abce1*, *Atp6ap2*, and *Zeb2*. The Hitomi et al^31^. screen failed to identify *Ripk3* and *Mlkl*, suggesting that RNAi off-target activity or incomplete gene depletion may have prevented discovery of other necroptosis mediators. Importantly, treatment with exogenous TNFα in our screen avoids identification of genes required for production of autocrine TNFα, whereas the Hitomi et al^31^. screen identified c-Jun, which is required for TNFα transcription in L929 cells^23^.

Among the extended list of 59 novel screen hits that show moderate to no effect on normal cell viability (Table S3), some are known to function in common biological pathways and processes, suggesting related mechanisms-of-action (Table 1). Like *Tnfrsf1a*, *Ripk1*, *Cyld*, and *Spata2*, five other screen hits reportedly play various roles in inflammatory and apoptotic signaling. Notably, N4BP1 binds and inhibits the anti-inflammatory and pro-apoptotic ubiquitin E3 ligase ITCH by blocking its interaction with other substrates^32^. Since ITCH can form ubiquitin-editing complexes with the RIPK1 regulators CYLD or A20^8,33–37^, there are multiple putative mechanisms for N4BP1 functioning through ITCH to mediate necroptosis. Protein kinase OXSR1 and its direct activator kinase WNK1 were also identified as necroptosis resistance screen hits. This pathway functions in cell osmoregulation to increase water influx and cell volume by both activating Na^+^–K^+^–Cl^−^ cotransporters and inhibiting K^+^–Cl^−^ cotransporters^38–40^. Blocking this pathway may counteract cation influx and cell swelling induced by MLKL and plasma membrane permeabilization to prevent necroptotic cell death.

**Table 1:** Potential common mechanisms-of-action identified by new necroptosis regulators

Gene class	Gene (alias)	Gene functions^a^	Protein class or domains^a^
Inflammatory and apoptotic signaling	*N4bp1*	Substrate and inhibitor of pro-apoptotic and anti-inflammatory ITCH, which destabilizes RIPK1 (through A20) and TAK1 (through CYLD)	KH (RNA binding), UBA (ubiquitin interaction), and NYN (RNAse) domains
	*Traf1*	Dimerizes with TRAF2 to mediate TNFR1 activation of JNK, NF-κB, and anti-apoptotic signals	Adaptor protein
	*Nfkbia (IκBα)*	Blocks nuclear import of NF-κB/Rel, and Bax recruitment to VDAC1	Ankyrin repeats
	*Usp9x*	Stabilizes ITCH, MCL1, and XIAP	Deubiquitinase
	*Ube2k (Hip2)*	Promotes TNFα-mediated NF-κB activation; destabilizes Smac/DIABLO, p53, and Rb	Ubiquitin-conjugating enzyme
WNK-OSR1/SPAK signaling	*Oxsr1 (Osr1)*	Activates Na(+)-K(+)-2Cl(-) cotransporters; inhibits K(+)-Cl(-) cotransporters; osmoregulation; ischemic neuronal cell death	Ser/Thr protien kinase
	*Wnk1*	Activates OXSR1 and paralog STK39/SPAK; cell volume recovery during osmotic stress; inhibits autophagy	Ser/Thr protein kinase
Lysosome-endosome-Golgi-ER trafficking and function	*C030046E11Rik (Ric1)*	Retrograde transport from endosomes to Golgi; Ric1-Rgp1 complex is GEF for late Golgi Rab6A GTPase	WD40 repeat
	*Rgp1*	Retrograde transport from endosomes to Golgi; Ric1-Rgp1 complex is GEF for late Golgi Rab6A GTPase	
	*Cog1 (Ldlb)*	Intra-Golgi vesicle, endosome-Golgi, and Golgi-ER retrograde transport to maintain Golgi structure and function	
	*Cog3 (Sec34)*	Intra-Golgi vesicle, endosome-Golgi, and Golgi-ER retrograde transport to maintain Golgi structure and function	
	*Cog4 (Cod1)*	Intra-Golgi vesicle, endosome-Golgi, and Golgi-ER retrograde transport to maintain Golgi structure and function	
	*Cog6 (Cod2, Cdg2l)*	Intra-Golgi vesicle, endosome-Golgi, and Golgi-ER retrograde transport to maintain Golgi structure and function	
	*Arfrp1 (Arp1, rl18)*	Retrograde transport from endosomes to Golgi; Golgi-to-plasma membrane transport	Plasma membrane-associated GTPase
	*Vps29*	Retrograde transport from endosomes to Golgi or plasma membrane; retromer complex component	Metallophosphatase superfamily (inactive)
	*Tmed9 (Gmp25, p24alpha2)*	Cargo receptor for Golgi-ER retrograde transport (COPI complex component); promotes ER exit of proteins	
	*Sec23ip (p125A)*	ER-Golgi anterograde transport (COPII complex component)	Phospholipase A1 family; SAM domain
	*Atp6ap2 (prorenin receptor)*	Stabilizes V-ATPase; acidification of endodomes and lysosomes; endolysosomal protein sorting and degradation; adaptor between V-ATPase and Wnt receptor complex	
	*Atp6ap1 (Ac45)*	Promotes V-ATPases activity; acidification of endodomes and lysosomes; calcium-dependent membrane fusion	
Nonsense-mediated mRNA decay (NMD)	*Smg7*	NMD; stabilizes p53 (promotes ATM phosphorylation of MDM2)	Est1 DNA/RNA binding domain
	*Smg8*	NMD	
	*Upf2*	NMD; mRNA nuclear export	
	*Xrn1*	Decapped mRNA degradation; NMD	Exonuclease
	*Ddx6 (Rck)*	DCP1 mRNA decapping complex activator; mRNA degradation, translation, and/or translational repression	DEAD-box helicase
mRNA splicing	*Ptbp1*	Alternative splicing factor; binds mRNA polypyrimidine tracts	RRM (RNA binding) domains
	*Pcbp1*	mRNA processing; binds mRNA poly(rC); iron chaperone for metalloproteins	KH (RNA binding) domains
	*Mettl3*	N6-methyladenosine [m(6)A]:RNA methyltransferase; regulates splicing and mRNA export, translation, and decay	
	*Ythdc1 (Yt521)*	mRNA methylation [m(6)A] reader protein; alternative splicing factor	YTH [m(6)A-binding] domain

^a^Annotations are derived from references cited in the text, or from Gene [Internet] (Bethesda, M): National Library of Medicine, US, National Center for Biotechnology Information; 2017 Jul 09. Available from: https://www.ncbi.nlm.nih.gov/gene/)

While retrograde transport of proteins from early endosomes to the trans-Golgi network (TGN) has not previously been implicated in necroptosis, our screen identified eight mediators of this pathway, including both subunits of the RAB6A guanine nucleotide exchange factor (RIC1 and RGP1)^41^ and four subunits of the component of oligomeric golgi complex (COG1, COG3, COG4, and COG6)^42^. Endosome-to-Golgi retrograde transport returns sorting factors from the endo-lysosomal system to the TGN for reuse, thereby maintaining TGN functions of endosome, lysosome, and secretory vesicle biogenesis^43,44^. Two other screen hits, ATP6AP1 and ATP6AP2, are accessory subunits of the vacuolar (H^+^)-ATPase (V-ATPase) proton pump that mediate acidification of endosomes and lysosomes to maintain their protein sorting and degradation activities^45^. ATP6AP2 (prorenin receptor) also functions as an adaptor between the Wnt receptor and V-ATPase complexes and, along with V-ATPase activity and receptor endocytosis, is required for Wnt/β-catenin signaling^46,47^. TNFR1 complex is internalized into endosomes upon TNFα binding, where CASP8 and other components are recruited and apoptotic signaling is initiated^48^, and can be subsequently routed to the lysosome and TGN, although it is not thought to recycle back to the plasma membrane^49^. We speculate that proper endosome composition and acidification may be required for internalized TNFR1 complex maturation and subsequent necrosome formation.

Another common pathway among five screen hits is nonsense-mediated mRNA decay (NMD) that recognizes and degrades transcripts containing premature termination codons, as well as normal mRNAs with certain other structural features^50^. NMD can selectively target specific mRNAs to suppress the unfolded protein response, oxidative stress response, autophagy, and apoptosis, but has not previously been implicated in regulating necroptosis. Four screen hits are known to regulate mRNA splicing, although through the different transcript features of N6-methyladenosine (METTL3 and YTHDC1)^51^, poly(C)-rich regions (PCBP1)^52^, or polypyrimidine tracts (PTBP1)^28–30^. RIPK3 alternative splice products that abolish activity have been identified^53^, although a regulatory role in necroptosis has not been defined.

We demonstrated that the PTBP1 alternative splicing factor specifically maintains expression of RIPK1, and not other necrosome components, by inhibiting inclusion of a cryptic exon that would alter the reading frame, introducing a translation termination codon upstream of the kinase domain. *Ptbp1* knockout strongly reduces *Ripk1* transcript and protein levels, presumably through mRNA NMD. PTBP1 and its paralogs PTBP2 and PTBP3 mediate alternative splicing programs during development of multiple tissues through direct binding to mixed pyrimidine tracts in nascent pre-mRNA to repress splice site recognition, by competing with U2AF binding to the 3′ splice site as well as additional mechanisms^54^. Most PTBP1-repressed exons are cassette exons that are skipped and contain a CU-rich tract in the intron adjacent to the 3′ splice site^29^, and we have shown this to be the case for *Ripk1*. A subset of these alternative splicing events also appear to prevent inclusion of a premature stop codon and transcript downregulation by NMD. We have shown that the alternative exon deleterious to RIPK1 protein expression is conserved in the human *RIPK1* gene and similarly regulated by PTBP1. PTBP1-dependent *Ripk1* alternative splicing does not depend on the induction of necroptosis, and the alternative splice isoform is normally expressed at detectable levels in a variety of human and mouse tissues. Interestingly, inclusion of the *RIPK1* deleterious exon increases during differentiation of iPSC-derived neural stem cells as *PTBP1* levels decline to reprogram the splicing pattern of many genes toward a neuronal cell state. We speculate that this mode of regulating RIPK1 availability might be utilized by some developmental, tissue-specific, or stress response pathways to allow or prevent regulated cell death.

## Materials and methods

### Cell culture

L929 (CCL1-1, ATCC), HT-29 (HTB-38), and COLO 205 (CCL-222) cells were modified to express human codon-optimized Cas9 introduced using the blasticidin selectable pLENTI6.3 Cas9^55^. L929.pLENTI6.3-Cas9 cells were cultured in Dulbecco’s modified Eagle’s medium (DMEM) supplemented with 10% fetal bovine serum (FBS; F6178, Sigma), 2 mM Glutamax (35050061, ThermoFisher), 100 IU penicillin, and 100 μg/ml streptomycin (15140122, Thermofisher), 1% non-essential amino acids (11140050, ThermoFisher), 10 mM HEPES pH 7.2 and 6 μg/ml blasticidin (A1113903, ThermoFisher). HT-29.pLENTI6.3-Cas9 and COLO 205.pLENTI6.3-Cas9 cells were cultured in RPMI1640 supplemented with 10% FBS and 10 μg/ml blasticidin. HEK-293T cells were cultured in DMEM supplemented with 10% FBS, 2 mM GlutaMAX, and 1% non-essential amino acids. Human iPSC-derived neural stem cells (MTI-Global Stem, GSC-4311) were maintained in NSC maintenance medium (DMEM/F12/Neurobasal, 1× GS22, 20 ηg/ml bFGF, 20 ηg/ml EGF, 20 ηg/ml BDNF, 0.11 mM β-mercaptoethanol, 0.5 mM GlutaMAX) and differentiated in NSC differentiation medium (DMEM/F12/Neurobasal, 1× GS21, 1× N2, 5 μg/ml cholesterol, 1 mM creatine, 100 μM ascorbic acid, 0.5 mM cAMP, 20 ηg/ml GDNF, 20 ηg/ml BDNF, 1 μg/ml laminin, 0.5 mM GlutaMAX). iPSC neural conversion was monitored by microscopic inspection of cells.

### sgRNA sequence design

RefSeq sequences were downloaded on June 25, 2014, and transcript models identified using GMAP^56^ and Splign^57^ alignments to UCSC mm10. RefSeq CDS annotations were used to determine coding regions. Candidate guide 19-mers with an NGG PAM that were entirely contained in a CDS (including the PAM) and were unique in the genome with respect to both NGG and NAG PAMs were collected. A local version of the MIT algorithm^58^, modified to evaluate only NGG PAMs because of run-time constraints, was used to estimate off-target scores. Sequences with GC content <15% or >85%, four Ts or three consecutive Ts in the five PAM-proximal positions, four or more consecutive Gs, or homopolymers of five or more bases were eliminated. Eight guides with off-target scores greater than or equal to 70, hitting the most isoforms for each gene, and with a preference for the 5′ end were selected, resulting in 98.1% of genes having eight or more guides. For 630 genes tagged as “low-confidence”, some restrictions were relaxed in order to obtain at least three guides. Positive controls consisted of 58 genes for which 12 guides were designed and 47 NTC with low similarity to the mouse genome (Table S1).

### Pooled CRISPR library design and lentivirus preparation

In total, 155,120 guides targeting 19,883 mouse genes were optimally selected using a custom sgRNA design algorithm (Table S1; see Extended Methods). The sequences were produced in nine arrayed oligo synthesis pools (Cellecta, Inc.) and sub-cloned into a puromycin selectable lentivirus vector pLKO_SHC201 (Sigma) termed “library vector” in the subsequent text. The combined packaging transfection mix comprised lentivirus library vector, pCMVdR8.9 (expressing gag, pol, and rev genes) and pCMV-VSV-g (expressing envelope protein). Lentivirus libraries were assessed for quality by transducing RKO cells (CRL-2577, ATCC) with serial dilutions of the virus and counting puromycin-resistant cells 5 days post transduction. NGS analysis of sgRNA reference libraries obtained through large-scale transduction of RKO cells confirmed the individual guide proportions to be consistent with the manufacturer’s specifications.

### Infectious virus titer determination

In total, 1.11 × 10^5^ cells in a volume of 1 ml were seeded in six-well dishes and incubated overnight at 37 °C, 5% CO_2_. The virus stock was first diluted 1/40 followed by four five-fold serial dilutions in ice-cold medium containing 48 μg/ml polybrene (TR-1003-G, EMD Millipore) as the virus diluent (for a final concentration of 8 μg/ml). In total, 0.2 ml of each virus suspension was used to infect five wells, while, a sixth well was treated with 0.2 ml of virus diluent alone. Plates were returned to the incubator for 5 h incubation after which time each well was replenished to 2 ml with culture medium and incubated a further 48 h. Cells from each virus dilution infection well were harvested, and 10,000 cells in 100 μl were re-plated to a 96-well plate. Following overnight incubation, wells were treated with 12.5 μl of 72 μg/ml puromycin medium (A1113803, Thermofisher) (for a final concentration of 8 μg/ml), and replicate wells were treated with an equivalent volume of medium. After 72–96 h treatment, the proportion of infected, puromycin-resistant cells was determined by a viability assay using the CellTiter-Glo method, and infectious virus titer was calculated based on the virus volume used (see Supplementary Information Extended Methods).

### Screen set-up

Cells were plated the day before transduction into color-coded vessels to distinguish replicates for processing as three individual experiments that were strictly kept separate for the duration of the cell cultivation process and through NGS analysis (Figure S10). The first of the sub-libraries, which targets the subset of genes within “Library 1”, was screened as a proof-of-concept necroptosis resistance positive selection screen. Library gene members and targeting sequences are available in Table S1. L-929.pLENTI6.3-Cas9 cells were infected with a virus dose to achieve a MOI of 0.3, with sufficient cell numbers plated to obtain a screening depth of 1000 cells per sgRNA, accounting for the estimated proportion of uninfected cells under a Poisson probability distribution. sgRNA representation for the remaining libraries was varied to increase throughput (see Supplementary Information Extended Methods).

### sgRNA quantification

gDNA was isolated using Puregene reagents (158788, Qiagen). DNA concentration was measured using a Qubit assay (Q32853, Molecular Probes). To quantify the abundance of sgRNAs, a gDNA amount consistent with the screening sgRNA representation was used as template for sgRNA amplicon generation by PCR using primer sequences in common with all sgRNAs (see Supplementary Information Extended Methods). NGS read counts of the required depth were obtained and processed using gCrisprTools^24^, a modification to previous published methods^59^. sgRNA fold-change relative to the control reference (day 2) or to control expansion (day 15) samples was calculated from size factor-adjusted, median sgRNA-scaled raw reads, and then log_2_ transformed.

### Library screen and hit validation micro-scale necroptosis assays

Necroptosis in L-929.pLENTI6.3-Cas9 cells was induced through media supplementation with murine TNFα (MRRD55, Genentech) to 100 ηg/ml combined with Z-VAD-FMK (G7572, Promega) to 20 μM (TZ) for 12–16 h, whereas HT-29.pLENTI6.3-Cas9 and COLO 205.pLENTI6.3-Cas9 cells were pre-treated with 200 μM Z-VAD-FMK for 2 h followed by treatment of 10 ηg/ml TNFα combined with 1 μM BV6 (TZB) for 12–16 h. Necroptosis resistance was assayed in the large-scale screens by sgRNA count enrichment (Table S1), and in the micro-scale assays by the CellTiter-Glo (Promega) viability assay (comparing TZ or TZB treated with untreated cultures; see Extended Methods).

### crRNA transfection and lentivirus sgRNA transduction

L-929.pLENTI6.3-Cas9 cells were transfected with crRNA:tracrRNA hybrids at 25 nM in 0.25% RNAiMax (percent by volume). Successful Cas9-mdiated gene knockout was monitored through visualization of morphology changes for a Cdk1 transfection control. HT-29.pLENTI6.3-Cas9 and COLO 205.pLENTI6.3-Cas9 cells were infected with lentivirus containing sgRNA expression constructs in the presence of 8 μg/ml polybrene at varying MOI. TZ or TZB resistance was monitored to ensure successful control gene knockout. Necroptosis assays were performed at least 5 days after crRNA transfection.

### Western blotting

Cells were processed into lysates in ice-cold Phosphosafe buffer (71296, EMD Millipore) supplemented with dodecyl maltoside to 0.5% and complete protease inhibitor tabs (11 873 580 001, Roche). DNA was sheared by passing the lysates through a 23-G needle. Clarified lysates were normalized for protein content by BCA protein assay (23227, Pierce) prior to denaturation in LDS loading buffer (NP0007, ThermoFisher) supplemented with sample reducing agent (NP0009, Thermofisher), followed by protein separation and transfer to nitrocellulose membranes (see Extended methods). Membranes were blocked in TBS 5% milk for 2 h at room temperature. Proteins were immuno-detected by incubation with primary antibodies overnight at 4 °C, followed by compatible species-specific secondary infrared fluorescent dye-labeled anti-IgG solutions for 2 h at room temperature, and membrane scanning on an Odyssey imager (Licor) (antibodies are listed in Table S5).

### Real-time quantitative PCR assays

Total RNA was purified using a QIAcube (Qiagen) standard protocol, which includes the DNase digestion step. Mouse *Ripk1* transcript was quantified from 25 ηg L-929.pLENTI6.3-Cas9 total RNA, whereas human *RIPK1* was quantified from 50 ηg HT-29.pLENTI6.3-Cas9, COLO 205.pLENTI6.3-Cas9, or iPSC-derived cell total RNA as input to real-time quantitative PCR (RT-qPCR) reactions using Applied Biosystems TaqMan RNA-to-CT reagents (Thermofisher) in a CFX384 Touch Real-Time PCR Detection System (Bio-Rad). Canonically and alternatively spliced *Ripk1* RNA transcripts were detected with the gene expression assays listed in Table S6. ΔΔCt calculations relative to non-targeting control treatments were used to determine expression levels^60^. Tissue mRNA expression was surveyed using human cDNA arrays (HMRT104 and HBRT101, OriGene) and mouse cDNA arrays (MDRT101 and MNRT101, OriGene) by RT-qPCR according to the manufacturer’s instructions.

## Electronic supplementary material

Supplementary Information

Figure S1

Figure S2

Figure S3

Figure S4

Figure S5

Figure S6

Figure S7

Figure S8

Figure S9

Figure S10

Table S1

Table S2

Table S3

Table S4

Table S5

Table S6

Table S7
