# Supplementary material for: CRISPR whole-genome screening identifies new necroptosis regulators and RIPK1 alternative splicing
Source: Cell Death Dis. 2018 Feb 15;9(3):261. doi: 10.1038/s41419-018-0301-y (PMC5833675; doi:10.1038/s41419-018-0301-y)
Supplement: Supplementary file 1 — Supplementary Information [file 41419_2018_301_MOESM1_ESM.docx]

**Supplementary Information**

**Extended Methods**

**Library virus preparation**

Each sub-library plasmid pool was packaged by co-transfecting 613.2 μg library plasmid DNA (in vector pLKO_SHC201), pCMVdR8.9 (expressing gag, pol and rev genes) and pCMV-VSV-g (expressing envelope protein) in the molar ratio of 1:2:0.2 using Lipofectamine 2000 reagent (11668019, ThermoFisher). The transfection mix was distributed over fourteen 15-cm cell culture plates containing 2.7x10^7^ HEK293T cells each plate.  6 h post-transfection, the medium was aspirated and replaced with 30 ml of fresh growth medium supplemented with 1 U/ml DNase I (79254, Qiagen), 5 mM MgCl_2_ and 20 mM HEPES pH7.2. After overnight incubation at 37°C, the medium was aspirated and replaced with 30 ml of fresh growth medium. After another 24 h incubation, the lentivirus-containing medium was collected and pooled, clarified by filtering through a 0.45 μm bottle-top filter, and concentrated using the Lenti-X Concentrator (631231, Clontech). Concentrated lentivirus was re-suspended in PBS with 1% BSA, aliquoted, frozen in dry ice/ethanol, and stored at -80°C.

**Puromycin resistance determination by CellTiter-Glo (CTG) method and titer calculation**

CTG assays were developed by the addition 28 μl 5X CTG reagent (reconstituted in 40 ml of lysis buffer) to 112.5 μl in a 96-well plate and incubated for 15-20 min before recording luminescence. The proportion of infected cells was calculated as (CTG counts with puromycin treatment) / (CTG counts without puromycin treatment). CTG counts were used to calculate for each dilution the infectious units concentration (IFU/ml) present in each virus sample tested: (proportion of infected cells) X (1.11x10^5^ cells plated) / (volume of virus for infection). The range of virus volumes tested was plotted against the calculated IFU, and either a point-to-point curve or a fitted curve was used to interpolate the IFU (x-axis) and volume (y-axis) at the most linear part of the curve to arrive at the IFU/μl (infectious titer) for the virus stock.

**Library screen virus infection**

5.25x10^6^ L929.pLENTI6.3-Cas9 cells were plated into each of 13 T-225 flasks per individual experiment (assigned pink, grey and blue). Cell numbers for infection were calculated using the formula (17214 library sgRNAs) X (1000 sgRNA representation) / (1 – e^-MOI^) and distributed to the number of flasks needed to accommodate a suitable cell density for 3 days of sub-confluent growth. The following day, the media in T-225 flasks was reduced to 20 ml and virus was added in 4 ml of virus diluent. Flasks were returned to the incubator for 5 h, after which time medium was replenished to 40 ml 48 h post infection. Two days after transduction, cells from each experiment were collected by trypsinization, a minimum of 6.62x10^7^ cells were flash frozen for later gDNA extraction, and the remainder returned to culture. The following day, cells were placed under selection with 8 μg/ml puromycin. For increased throughput, the MOI and sgRNA representation were modified for subsequent library screens (Table S7).

**Library screen and micro-scale necroptosis assays**

Cells recovered from the screening replicate pools were stratified into two experimental arms, maintaining paired replicates: ‘Death expansion’ and ‘Control expansion’. The ‘Control expansion’ arm received sufficient cells to maintain the desired sgRNA representation, whereas the ‘Death expansion’ arm received the majority of cells. Also at this time, a sample of 10,000 library screen cells was plated into each well of a 96-well plate for a micro-scale cell death assay. The spent medium from the cell harvest was filtered to remove any cell debris and reserved for post-treatment regrowth of necroptosis resistant cells. 24 h after cell plating, the ‘Death expansion’ was subjected to necroptotic stimuli through the addition of 5 ml of 9X concentrated death cocktail comprised of 900 ηg ml^−1^ TNFα and 180 μM zVAD (TZ) to each 40-ml treatment flask, while the ‘Control expansion’ arm received no treatment. In parallel, half the wells in the micro-assay plate were treated with the same preparation (*i.e*., 12.5 μl death cocktail added to 100 μl of plated cells), while the other half received an equivalent amount of medium. After 12 to 16 h treatment, death cocktail was exchanged for spent medium, and the proportion of cell killing was determined by the assaying cell viability in the 96-well micro-assay by the CTG method: 1 – [(CTG counts with TNFα+zVAD treatment) / (CTG counts without treatment)]. Screen hit validation micro-scale assays where performed in a variety of plate formats explained in detail below using optimized necroptosis-inducing cocktails specific to the cell type.

**sgRNA quantification**

Multiple 2-μg genomic DNA PCR reactions sufficient to maintain library complexity were used for amplification with sgRNA-specific primer pairs TCTTGTGGAAAGGACGAGGTACCG (gRNA_Forward) and TCTACTATTCTTTCC CCTGCACTGT (gRNA_Reverse). After an initial denaturation at 98°C for 3 min, thermal cycling proceeded with 28 cycles at 98°C for 10 sec, 56.9°C for 30 sec, and 72°C for 20 sec (Proflex PCR system, ThermoFisher). Amplicon cleanup was performed using 1.8X AMPURE XP beads (A63882, Beckman Coulter) with a Biomek FX automated binding, washing, and elution procedure. A second round of PCR was performed for NGS library construction using100 ηg of the first-round PCR DNA for each experimental replicate using Nugen Ovation Library System for Low Complexity Samples. Paired-end sequencing was carried out on Hiseq 2500 (Illumina) in Rapid mode (2x150 cycles). gCrisprTools was employed for quality control and analysis of NGS reads.^1^

**Library screen data processing**

After using custom scripts to count the number of reads containing each sgRNA sequence in each sample for each sub-library screen, we independently analyzed each experiment using the gCrisprTools software package with the default parameters.^1^ Briefly, after discarding low-abundance sgRNAs in the reference samples and median scaling the remaining sgRNA distributions, we quantified the evidence for differential sgRNA construct abundance between the treated and untreated samples in the framework of a precision-weighted linear model using the expression estimates and weights returned by voom.^2^ We performed this as a direct pairwise comparison between the treated and untreated endpoints, equivalent to an unpaired moderated t-test performed on the expression estimates and incorporating the precision weights. The P-values and coefficient estimates associated with each sgRNA were then aggregated in the RRAα framework (α = 0.1)^3^ to generate gene-level significance estimates (gene-level *P*-values). These gene-level *P*-values are permutation-based and consequently lose resolution among the most significantly enriched gene candidates, so we used the Rho statistics calculated within RRAα framework (parametric statistics directly related to the gene-level *P*-values)^4^ to rank top candidates for validation. We note that this analytical approach is fundamentally based on the gRNA signal rankings within each screen, which are in turn dependent on the effects of the other gRNAs that are present within the library or libraries used. Consequently, while the Rho and *P*-value estimates that we report are valid within each screen, we recommend that readers use caution when comparing these values across distinct experiments.

**crRNA transfection and lentivirus sgRNA transduction**

L929.pLENTI6.3-Cas9 cells were seeded for crRNA transfection at 62,500 cells per well in 6-well plates for RNA extraction, and at 25,000 cells per well in 12-well plates for protein detection. In parallel, the same crRNA transfection mixes were applied to cells plated at 2,500 cells per well in 96-well plates to monitor necroptosis resistance conferred by specific gene knockout. HT-29.pLENTI6.3-Cas9 and COLO 205.pLENTI6.3-Cas9 lines were seeded at 5,000 cells per well in 96-well plates and infected with lentiviral sgRNAs (targeting sequences are listed in Table S4). Cells were passaged into selection medium containing 2 μg ml^-1^ puromycin. The infections that gave the greatest cell survival in puromycin were expanded serially as replicate cell pools in 12-well plates and tested at each of four passages for necroptosis resistance, ensuring effective Cas9 editing of control genes prior to RNA extraction for RT-qPCR assays.

**Real-time quantitative PCR (RT-qPCR) assays**

L-929.pLENTI6.3-Cas9 cells were transfected with crRNA:tracrRNA targeting *Ptbp1* and other necrosome components, and then monitored for at least 5 days prior to RNA extraction. RNA from HT-29.pLENTI6.3-Cas9 and COLO 205.pLENTI6.3-Cas9 cells infected with lentiviral sgRNA targeting *PTBP1* and controls was extracted from puromycin-selected cells in 12-well plates. RNA from NSCs was extracted from cells plated onto poly-D-lysine- and laminin-coated 6-well plates at 70,000 cells/cm^2^ in NSC differentiation medium with half of the medium exchanged for fresh medium every 3-4 days for the duration of the experiment.

**Antibodies and Western blotting**

Transblot turbo (BioRad) was used for transfers to nitrocellulose membranes(1704159, Bio-Rad), except when detecting phosphorylated Serine345-Mlkl where the Criterion blotter (Bio-Rad) was used with SWIFT transfer buffer (786‐373, G-Biosciences) containing 20% methanol. Primary and secondary detection antibodies were diluted in Hikari A and B (02363, Nacalai), respectively, at the dilutions listed in Table S5. Filters were washed in between antibody exchanges three times for 15 min per wash in TBS, 0.05% Triton-X100 and rinsed in water prior to scanning. Raw TIFF images were processed in Adobe Photoshop preserving linearity of the signals.

**References**

1. Bainer R, Ratman D, Haverty P, Lianoglou S. gCrisprTools: Suite of functions for pooled CRISPR screen QC and analysis. R package version 1.4.0. *Bioconductor* 2017.
2. Law CW, Chen Y, Shi W, Smyth GK. voom: Precision weights unlock linear model analysis tools for RNA-seq read counts. *Genome Biol* 2014; **15**: R29.
3. Kolde R1, Laur S, Adler P, Vilo J. Robust rank aggregation for gene list integration and meta-analysis. *Bioinformatics* 2012; **28**: 573-580.
4. Li W, Xu H, Xiao T, Cong L, Love MI, Zhang F *et al*. MAGeCK enables robust identification of essential genes from genome-scale CRISPR/Cas9 knockout screens. *Genome Biol* 2014; **15**: 554.

**Supplementary Figure Legends**

**Figure S1.**  Functional validation of L929 Cas9 stable cell line. (a) Western blot time course analysis of Mlkl protein knockout after sgRNA transduction. (b) Cas9, RIPK1, and RIPK3 expression levels in the L929.Cas9 line. (c) Levels of necroptosis resistance conferred at 5, 8, and 12 days after transduction with Mlkl sgRNAs, with cell viability measured 6 to 6.5 h after TNFα+zVAD treatment. (d) Rescue of necroptotic cell morphology changes 1 h after TNFα+zVAD treatment by prior transfection with *Mlkl* synthetic crRNA. -, cells transduced with control sgRNA virus targeting firefly luciferase; NTC, non-targeting control. Molecular weight marker sizes are indicated in Kd to the right of Western blots (b, c).

**Figure S2.**  sgRNA read count and fold-change distributions for individual screens. Mean values for the 3 replicates are plotted. Positive and negative control sgRNAs are highlighted by the indicated colors.

**Figure S3.**  Correlation of sgRNA read count fold-change between replicate screen samples. Values for each sgRNA in the Library 1 screen are shown, with non-targeting, *Ripk1*, *Ripk3*, and *Mlkl* sgRNAs marked with the indicated colors. (a-c) sgRNA read count fold-change after necroptosis induction (producing 98.3% cell death) is plotted for each of the three replicate screens compared to the others.

**Figure S4.** sgRNA enrichment or depletion Rho statistics for all genes organized by chromosome position. (a) Gene-level log-transformed Rho values for sgRNA enrichment after TNFα+zVAD treatment compared to untreated samples, with genes showing Rho scores less than 10^-9^ marked in black and with positive control genes labeled. (b) Log-transformed Rho values for sgRNA depletion in the absence of necroptosis induction (day 15 untreated compared to day 2 reference samples), with genes possessing Rho scores greater than 10^-8^ marked in black.

**Figure S5.**  *Ptbp1* maintains *Ripk1* mRNA expression. (a) Cell survival conferred by transfected crRNAs targeting *Ptbp1* and control genes, with and without necroptosis induction by TNFα+zVAD (normalized to samples without crRNA). Error bars represent standard deviation (N=3). (b) *Ptbp1* mRNA knockdown levels produced by these crRNAs, as measured by qRT-PCR for a region internal to exon 5 and normalized to *Actb* expression levels. Error bars represent standard deviation (N=2).

**Figure S6.**  Sequence alignment of mouse and human *RIPK1* genomic regions across exons 4 and 5. Predicted protein translations are shown above (mouse) or below (human) the DNA sequence, with the translation of the alternative exon in red (as well as its continuation into exon 5 sequence for mouse). Exon nucleotides are in bold, and conserved nucleotides are highlighted in blue. C/T nucleotides are marked in pink for the putative PTBP1-binding polypyrimidine tracts. Premature termination codons introduced by the alternative splice are highlighted in red. *, stop codon.

**Figure S7.** *PTBP1* regulation of *RIPK1* splicing and necroptosis resistance. (a-d) Proportion of alternatively spliced *RIPK1* isoform mRNA relative to the canonical splice product, detected by qRT-PCR using primer and probe sets spanning exon 4 and the alternative exon (4-Alt in a and b; c) or the alternative exon and exon 5 (Alt-5 in a and b; d), after human HT-29 (a), human COLO 205 (b), or mouse L929 (c and d; treated with or without TNFα+zVAD as indicated) cells infection with the indicated sgRNAs or transfection with the indicated crRNAs. (e and f) *RIPK1* canonical splice isoform expression levels, detected by qRT-PCR using primer and probe sets spanning exon 4 and exon 5, after HT-29 (e) or COLO 205 (f) cell infection with the indicated sgRNAs. (g and h) Necroptosis resistance levels for HT-29 (g) or COLO 205 (h) cells infected with the indicated sgRNAs and treated with TNFα+zVAD+BV6, compared to untreated cells. Expression levels are normalized to human *RPLP0* (a, b, and e-h) or mouse *Actb* (c and d) mRNA detected in the same sample. Error bars represent standard deviation (N=3 for a, b, and e-h; N=2 for c and d).

**Figure S8.**  Expression of *PTBP1* and alternatively spliced *RIPK1* in human (a) and mouse (b and c) in the indicated tissues and developmental stages, detected by qRT-PCR using primer and probe sets spanning exon 4 and the alternative exon (4-Alt) or the alternative exon and exon 5 (Alt-5). Expression levels are normalized to human *RPLP0* (a) or mouse *Gapdh* (b and c) mRNA detected in the same sample and then, for PTBP1, to *PTBP1* in adult brain or, for *RIPK1*, to the canonical splice isoform of *RIPK1* (exon 4 spliced to exon 5) in the same tissue.

**Figure S9.**  *RIPK1* alternative splicing during neuronal differentiation of human iPSC-derived neural stem cells. *RIPK1, PTBP1, and PTBP2* mRNA expression levels during culture in NSC differentiation medium, detected by qRT-PCR using primer and probe sets spanning exons (4-Alt, exon 4 and the alternative exon; Alt-5, alternative exon and exon 5; 4-5, exons 4 and 5). Expression levels are normalized to *RPLP0* mRNA detected in the same sample. Error bars represent standard deviation (N=3).

**Figure S10.**  Diagrammatic workflow of screens. (a) Cells were passaged into flasks, harvested into tubes, and treated according to the time course shown for the paired replicates (N1, N2, and N3) of the screen arms (Control reference, Control expansion, and Death expansion). Replicates were maintained independently throughout the workflow. (b) Timeline of activities and cell numbers cultivated for Library 1 screen are portrayed in this example.
