# Supplementary figures and images for: CRISPR whole-genome screening identifies new necroptosis regulators and RIPK1 alternative splicing
Source: Cell Death Dis. 2018 Feb 15;9(3):261. doi: 10.1038/s41419-018-0301-y (PMC5833675; doi:10.1038/s41419-018-0301-y)

Figure S1

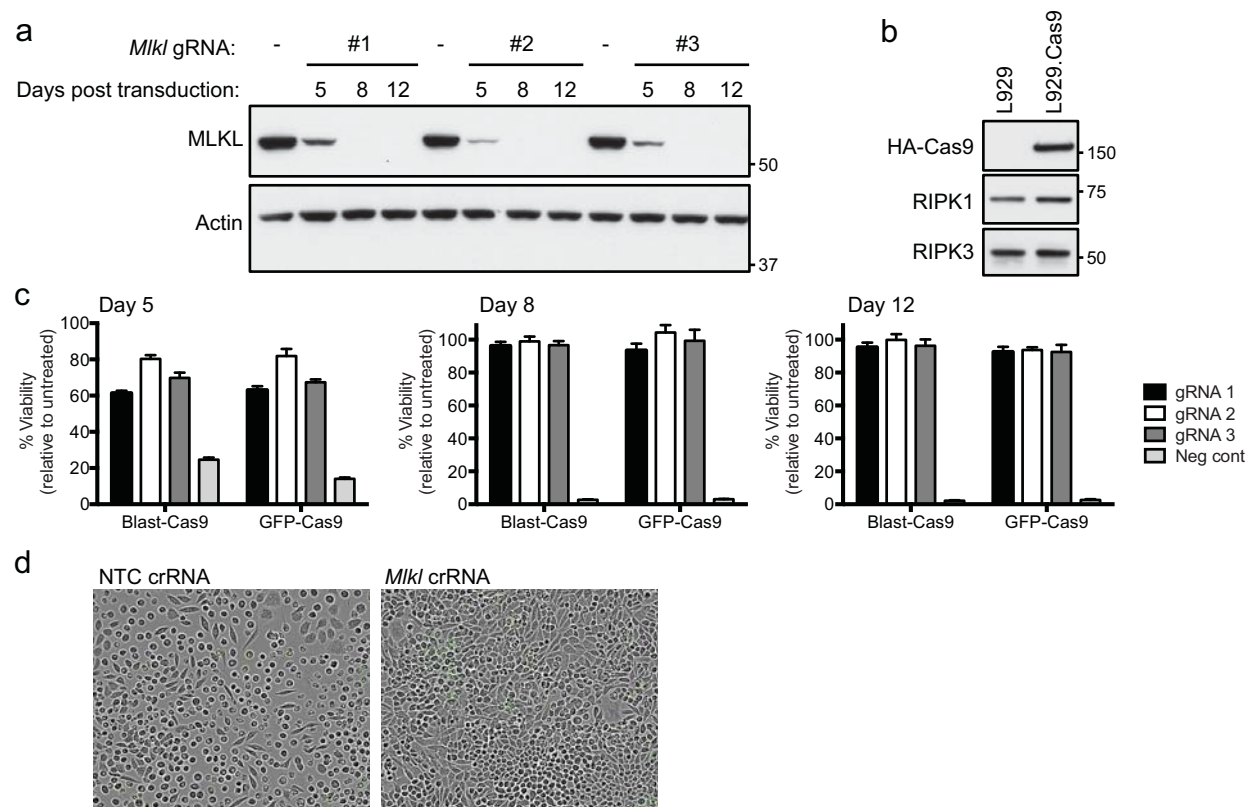

Supplement: Supplementary file 2 — Figure S1 [file 41419_2018_301_MOESM2_ESM.pdf]

Figure S2

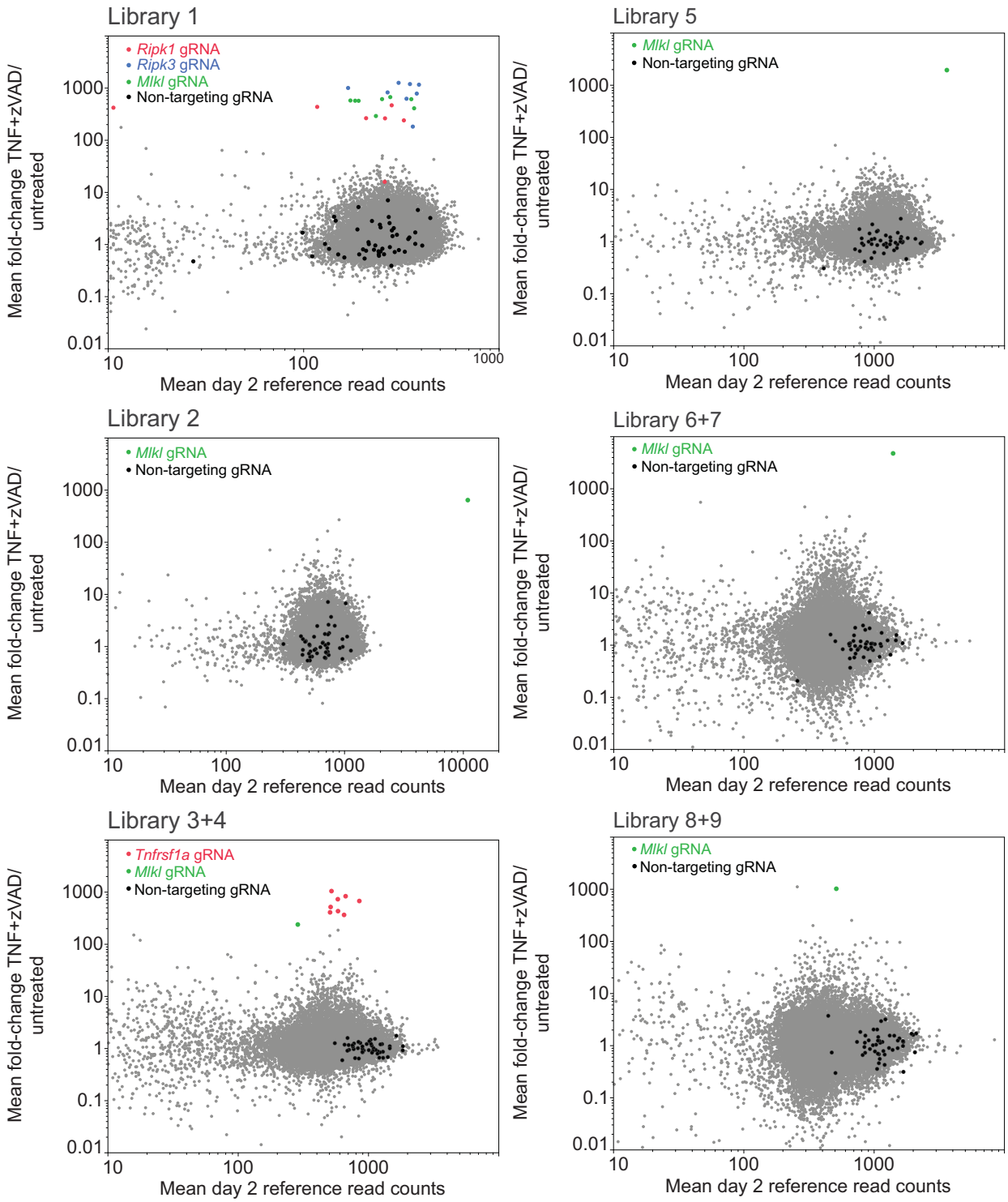

Supplement: Supplementary file 3 — Figure S2 [file 41419_2018_301_MOESM3_ESM.pdf]

Figure S3

**a**

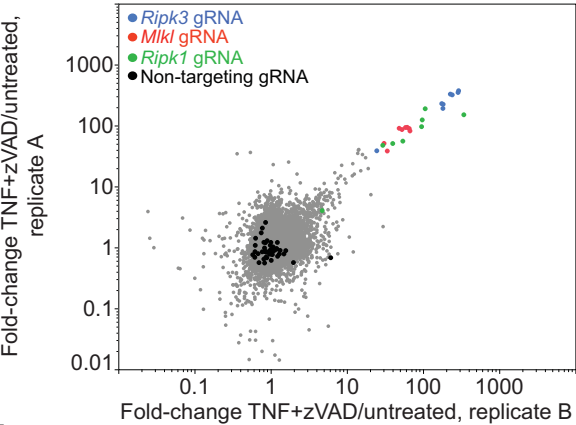

**b**

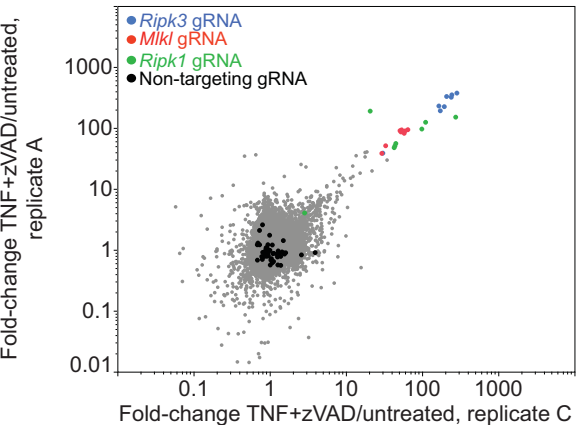

**c**

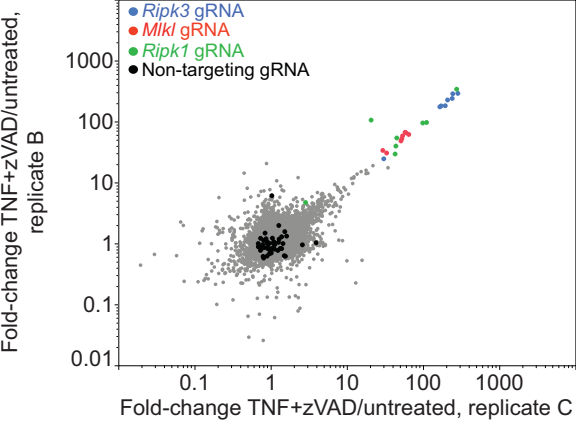

Supplement: Supplementary file 4 — Figure S3 [file 41419_2018_301_MOESM4_ESM.pdf]

Figure S4

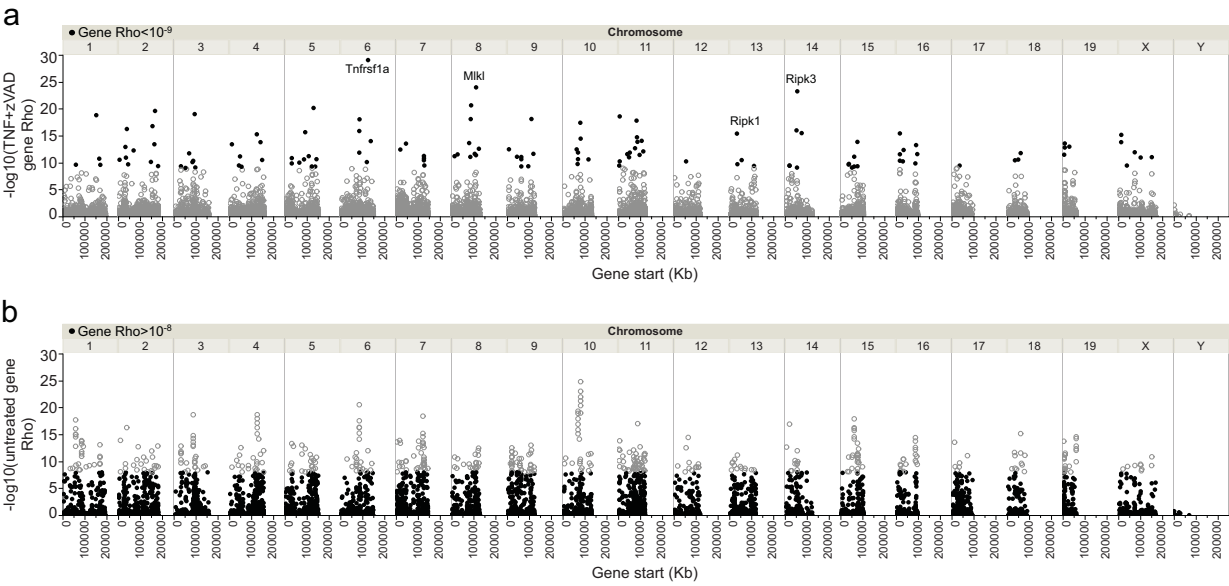

Supplement: Supplementary file 5 — Figure S4 [file 41419_2018_301_MOESM5_ESM.pdf]

Figure S5

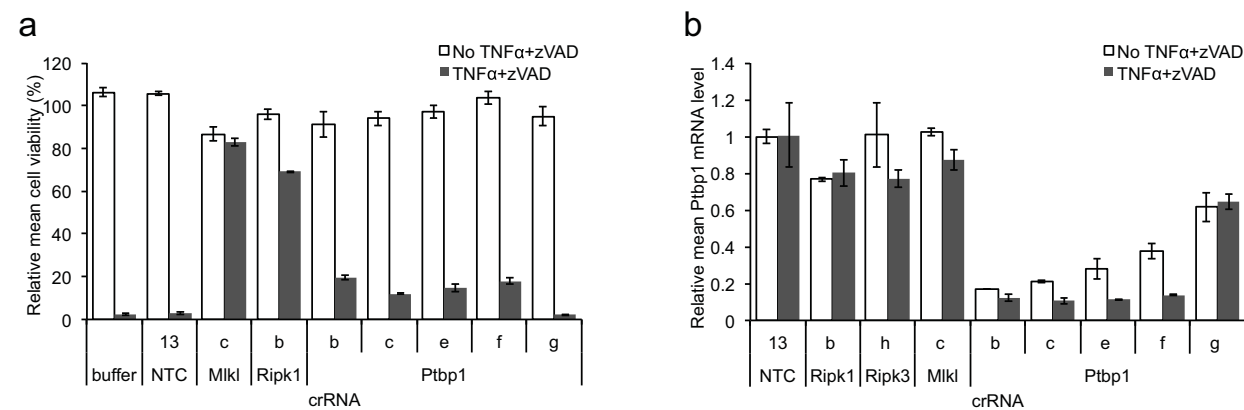

Supplement: Supplementary file 6 — Figure S5 [file 41419_2018_301_MOESM6_ESM.pdf]

Figure S7

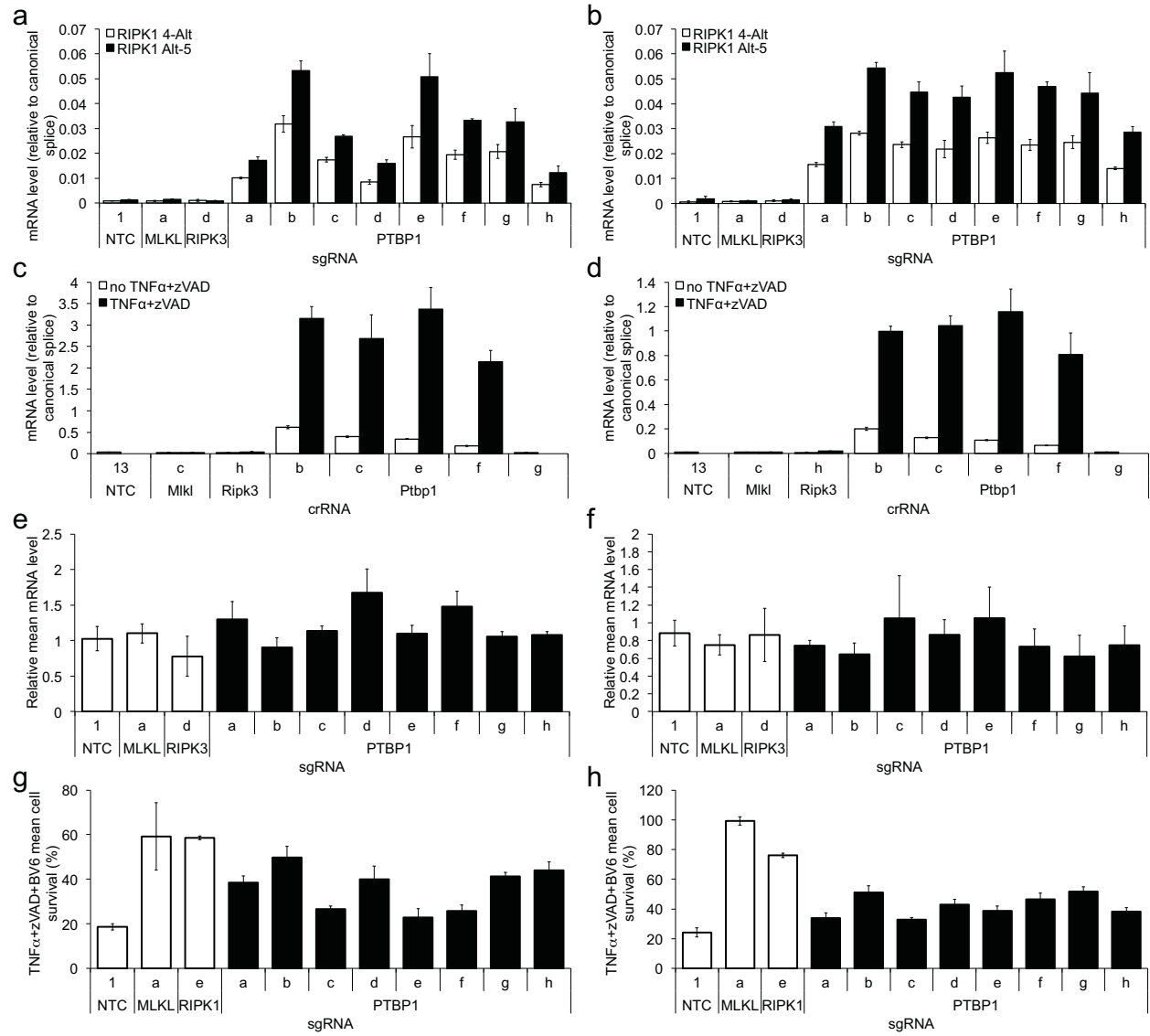

Supplement: Supplementary file 8 — Figure S7 [file 41419_2018_301_MOESM8_ESM.pdf]

Figure S9

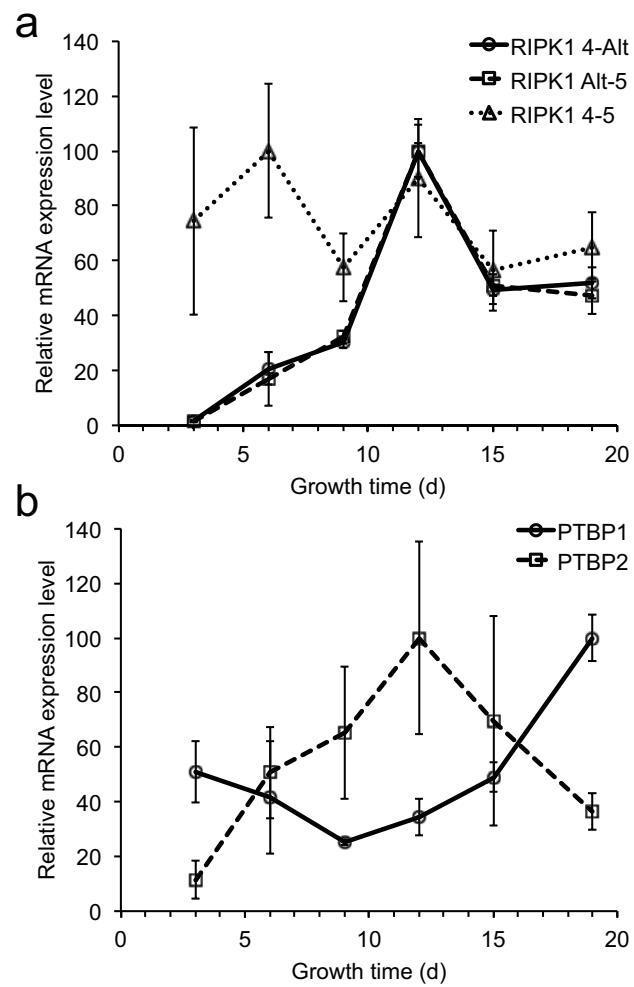

Supplement: Supplementary file 10 — Figure S9 [file 41419_2018_301_MOESM10_ESM.pdf]

Figure S10

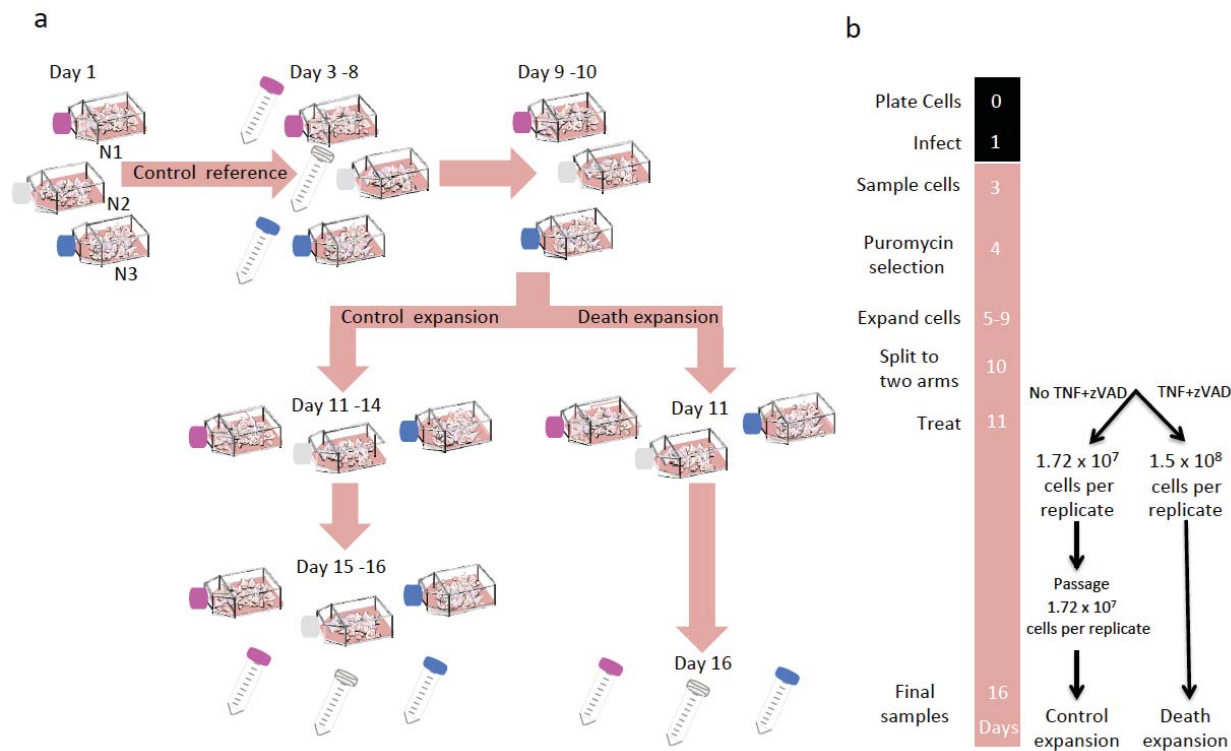

Supplement: Supplementary file 11 — Figure S10 [file 41419_2018_301_MOESM11_ESM.pdf]
