# Supplementary material for: CRISPR whole-genome screening identifies new necroptosis regulators and RIPK1 alternative splicing
Source: Cell Death Dis. 2018 Feb 15;9(3):261. doi: 10.1038/s41419-018-0301-y (PMC5833675; doi:10.1038/s41419-018-0301-y)
Supplement: Supplementary file 7 — Figure S6 [file 41419_2018_301_MOESM7_ESM.pdf]

Figure S6

```
Exon 4
                                                                                                                               I  D
Mouse  11521 GGAA-CCTGATAACACTAACCTTGGTCTCTCATCCCACC-CTCATGAGTGGAGTAATTGCTCTTGCCTGTTAGATTGCCTCTTCACTTCATAGACTTAATGTG--TTATTCCAGATAGAT
Human  11521 GGAAACCTGGTAACCTTCTCCTCAGGCTCAGATCCCACCACCCTTTCATGAAACAGTTGCTTTGGCCTATTTGATAACCTTTCCATTTCATAGACTTAATATCACTTGTTTTAGATGAGT
                                                                                                                               M  S

             V  P  L  S  L  K  G  R  I  I  V  E  A  I  E  G  M  C  Y  L  H  D  K  G  V  I  H  K  D  L  K  P  E  N  I  L  V  D  R  D
Mouse  11641 GTCCCACTTTCATTGAAAGGAAGGATAATCGTGGAGGCCATAGAAGGCATGTGCTACTTACATGACAAAGGTGTGATACACAAGGACCTGAAGCCTGAGAATATCCTCGTTGATCGTGAC
Human  11641 ACTCCGCTTTCTGTAAAAGGAAGGATAATTTTGGAAATCATTGAAGGAATGTGCTACTTACATGGAAAAGGCGTGATACACAAGGACCTGAAGCCTGAAAATATCCTTGTTGATAATGAC
             T  P  L  S  V  K  G  R  I  I  L  E  I  I  E  G  M  C  Y  L  H  G  K  G  V  I  H  K  D  L  K  P  E  N  I  L  V  D  N  D

             F  H  I  K
Mouse  11761 TTTCACATTAAGGTAATCCACAATCTGTATACTTTGAGAAGGCTGAATAATTGTAG-TTTAATTTTCCACTGGAAATCCATTCCTGATCAT-------GATGAATGCAGCATTCAG--CA
Human  11761 TTCCACATTAAGGTAAACCATCATCGGTAGGCTTCCAGAAAGTTGGGTGATTTTAGTTTTAATTCTCCAATGTAAATCCATTCTCGTACATTGGAGAAGCTAAAGGAAG-ACCTAGCTCA
             F  H  I  K

Mouse  11881 GCTTGCCACTGCTTGCAAGGCTGACA------------------TGGTATGAAAGGTGCAGGCTTGACTAGATCAAGAGAAGACTGTAAGGACA-----------------------CAG
Human  11881 GCTTATAACTGTTGATGATGGTGCCGAAAGGCTCTACCGGTGATTGAGAGGAAGGCTGCAGGCTTGATGGGATCAGGAGATAACTGTCAGGATAACTCATATCTTTTGGGGGAGGAGTGG

Mouse  12001 TGATGAA-----------AAAAAATCTCATCCCTGGTTCCTGGGTTTTGTTTCCTTCTGATTAGACTTAGAAA----TGGAAGCAGTCAAGGGTTGCTGTCTTCACTCTACAATGGGCTA
Human  12001 AGTTGAATGGGGTAGATGAGAAGACCTCTTTCCTAG--ACTGTGTT---TTTTCCTCTG-------TTAGAAACATTTAGAAACATTCAAGGGTTGCCCTTTTCATCCTGGAATGGGCCA

Mouse  12121 TCTCT-TCATCATTTTTCCCTGGGAATCT-TGTGACTAAGACTTCTCCACCCTGGAGATA-AAGGGAATATGGAAGTGTATGGAGAACATTCTTAGAATTCAGAAGTGGCTTAAAAGCAG
Human  12121 TTGGTGTCATAATGTTCTCCAGGAAATCTGTGTCACCAAGACTCTCCTCCTCTGGAAATGCAGGGGAATGTGAAGGTGTGTGCGGAAAATCCTTAGAGTTCAGATATGGCCTAAGAG...

Mouse  12241 CAGTAAATCAACCATTTTGGCCTCAGAATCCTCCACTTTGTGGAGCTGGAGGTATGGTTCAGCAGGGAATACCAGGCTTACACATCTGAGGTGCTAGGCTCAGACCCAGCACTCCAATCA
Human  12241 ....................................................................................................................ATCA

Mouse  12361 AAGACTTCTGCTTTGACATCCTGTTT-ATATCGATTTTC--------------------ATTTAAAAAGTCAATTTCCTTTTGAAA-ATTCTTTTTGTTATAGCAAAGATAATAGGGAAA
Human  12361 AAAACCTCTACTTTGAAATTTTGTTTTATATTTGTATTCGATTGTTTACTTCTGTCTGTATTTTAAAAATCAGTTTCCTTTGAACACATTCTTTCCATTGTCGTAAAGGGAACAAGGGAA

Mouse  12481 AGGACACCCATCTTTTGCCTATTCAGGGACTACATTTAACTGAGGAAGCAACAACACATATAATTAGTCTTGGTAGCTAGAATCCAAGCTGGAGA--CCTAAATGGGGACTAGATAGCTT
Human  12481 AGGG-ATTTTTCTTTTGTTCATTCAAGGGCAGGATTTAGCTAGGG--GGAGTGTCATATCTAATTGGCTTCTGCAGCAGGAATCCAAGCACAAGAATCCAAAAGGGGAAGCGGAAGG---

Mouse  12601 AGGTCAGAGTTCTGGGTTTCTTCCACAGACCTATACCAACAAGAAACAAAAAGAACCTGAGAACATGTAGCACTGAGGAAAAACCAGGACCTACCTACAAGACCCACATCAGACACC--G
Human  12601 -GGAAGCAGCCCTGGG--------------------------------GGAGGCACCCAGGAGCTGCTGACACAGGGG-----CCTCTCATCACCAGCAGGTTCTGCGTCTAGCGCTTTG

Mouse  12721 TTCATGTCTCAGA----------GTAATTAATCCCAGTGCCTTTCACCCTTTCAGTTCTGTTCATTCTTTCAATTAAGAACTTACCCTTTGTGATCTGGGTTAAAATCTGTGAATTTCAG
Human  12721 TCCTAGTCTTGGACAAAGGGCTGGGAATAAA----AATTCCTT----------ATTCATAACCTATCTTCAAATTCATGACTTCCCTCTTATGTCGTGGGTTAGAATCTGTGAATTTTAG

Mouse  12841 GTAAGCACATGCTCTAGCCAGCTCCTCCCTAAATGCTT---------CTTAGAGACAGAAGAGGACCATCTCCTTGGATTTTCCCAT-AACTGTCCTGCTCTGCCATTTTCCCTTGCCA-
Human  12841 GTACTCATGTGCCA----CAGTTCATCCCCACATGCTTGCTTGGATTCCTGGAGGCAGAAGTGGAACTGGCACCTATATTCTCTTCTGAACTGTGCTGCCCTGTCATTTTGCCCTGCCAG

Alternative exon
                                                                                                        V  Q  T  A  N  S  K  Q  V  A
Mouse  12961 CTTTCTTCTT-TGCTATGTCTGACTTGGTTATCTTTCTCT--GCCTTTATGTGTGTTTCCATTTCTTTCTCTCCATCCTGTCTTCTCTTAGGTTCAAACTGCCAACTCAAAACAAGTAGC
Human  12961 CACTGTTCTTGTTCTGTGTCTGACCTTGCTATTTTTCTCTCTGCTTTTTTGTCTGTTTT--TCTCCTCCTTTCCACCCTATCTCCTCTTAGGTTCAGGCTAC-AGCTCAAAACAAGCAAC
                                                                                                        V  Q  A  T   A  Q  N  K  Q  H

                A  T  I  S  P  S  G  W  L  W  D  A  R  K  N    S  H  R  P  L  D  A  I   G  Q  M  K
Mouse  13081 A--GCAACCATCTCACCCTCGGGGTGGCTTTGGGATGCTCGGAAAA--ACAGCCATCGTCCTCTGGATGCCATT-GGGCAAATGAAAGGTGTAGTAACCAGTCTCTATCTTCCCCAGAAA
Human  13081 ACTGCAGTC--CTTATCCTCAGTGTGTCTTTGGGATCCTTGGACACTTGCATCCATGGGCCTCTGGACGCCATTTGGGGAAATAGAAGGTGTAGTATTTAGCCACTGTCTGCTGCATAAA
               C  S  P    Y  P  Q  C  V  F  G  I  L  G  H  L  H  P  W  A  S  G  R  H  L  G  K  *

Mouse  13201 CAAAAACAAAAACAAAAACACATGCAAAGTAAATGAAAAATTAGTACAAACACCATTTTGTTGTCTTGCCCCAGTCTTATTGCTAAGAATTGAGATGTCAAGGCCCCACCCACCTGGTGA
Human  13201 TG--------------------TGCAGAG-----AAGAAAATA--------ACCATTTTCCCCCCAAGCCCTAGA---------AATAGTTGAAAAACATACGCAGAGTCAA--TGGGGA

Mouse  13321 ATGAAAAGAAAGGACTTCTGTAAGTTGTCCTCTGATTCACACACACATCTTGGCATATGTACTCCCCCCCCCTCCCGAAATACATAAATGTT----TAAAGAAACTTTAAAAAAAATAA
Human  13321 ATGAGAATAAATATGTTTGGTTGTTACTGTTCTTGT---CAGAAACATCTTC-------CGCTTTTCCCCTCCCCCTTACTGCATAAGGAATACGATACCAGAA--TTTCATGTGAACGT

Mouse  13441 TTGATATGCCAGTATTTTACA----------------TAAATATTCTGAGAATCTGTTGCAAGGTAGCTATTCAAGATAGGCCCCATGCAGCCA---CTGGAAAATTGATTGTGCCACAC
Human  13441 TTCCTGGGCGA-CATTTTACAAATGGCTGAGGAGACACACAGTTTCTATGAGTCTGTTGAGAAGTCGCTGTTTTGAACCTGCCCAGTGCAACCATTTCTGGAAAATTTACCGTACCTTAT

Exon 5
                                                                                                               I  A  D  L  G  V  A  S
                                                                                                                 S  R  S  W  C  G  F
Mouse  13561 ATGTACTGGATAAGACCCAGACTTGATTTCATGAAAATCCAGCTTAATTTACTTGTAGCCTTAGTGATA-ACTTCCATTGGCCCCTCTTCTTTTCCAGATAGCCGATCTTGGTGTGGCTT
Human  13561 GTATAATGGATAAGCCCAAAATT---------GAAAGTCAGATCTGATTTGCTTACGGCCTTCACCAAACAATCCCAGTGGCTCAATGTCTTTCGCAGATCGCAGACCTCGGCCTTGCCT
                                                                                                               I  A  D  L  G  L  A  S

               F  K  T  W  S  K  L  T  K  E  K  D  N  K  Q  K  E  V  S  S  T  T  K  K  N  N  G  G  T  L  Y  Y  M  A  P  E  H  L  N  D
              L  *
Mouse  13681 CCTTTAAGACATGGAGCAAACTGACTAAGGAGAAAGACAACAAGCAGAAAGAAGTGAGCAGCACCACTAAGAAGAACAATGGTGGTACCCTTTACTACATGGCACCCGAACACCTGAATG
Human  13681 CCTTTAAGATGTGGAGCAAACTGAATAATGAAGAGCACAATGAGCTGAGGGAAGTGGACGGCACCGCTAAGAAGAA---TGGCGGCACCCTCTACTACATGGCGCCCGAGCACCTGAATG
               F  K  M  W  S  K  L  N  N  E  E  H  N  E  L  R  E  V  D  G  T  A  K  K  N     G  G  T  L  Y  Y  M  A  P  E  H  L  N  D

               I  N  A  K  P  T  E  K  S  D  V  Y  S  F  G  I  V  L  W  A  I  F  A  K  K  E  P  Y  E
Mouse  13801 ACATCAATGCAAAGCCCACGGAGAAGTCGGACGTGTACAGCTTTGGCATTGTCCTTTGGGCAATATTTGCAAAAAAGGAGCCCTATGAGAGTAAGACATTTATAGTCACATGATTTTGAT
Human  13801 ACGTCAACGCAAAGCCCACAGAGAAGTCGGATGTGTACAGCTTTGCTGTAGTACTCTGGGCGATATTTGCAAATAAGGAGCCATATGAAAGTAAGGCATT....................
               V  N  A  K  P  T  E  K  S  D  V  Y  S  F  A  V  V  L  W  A  I  F  A  N  K  E  P  Y  E
```
